# Novel Isometric Testing of Frontal Plane Hip Strength in Collegiate Cross-Country Runners

**DOI:** 10.1055/a-2940-7121

**Published:** 2026-09-08

**Authors:** Tess Treinen Swake, Christopher M. Juneau, Jason Tuori, Gabriel J. Haberly

**Affiliations:** 1 Physical Therapy 5974 George Fox UniversityNewbergOregonUnited States; 2 Physical TherapyCJ Performance Solutions, LLCMiamiFloridaUnited States; 3 Physical TherapyJT Performance ConsultingNew YorkNew YorkUnited States

**Keywords:** hip strength, physical therapy, assessment, dynamometer, college athlete

## Abstract

In this cross-sectional study, we aimed to describe preseason hip abduction and adduction strength in Division III cross-country athletes using an externally fixated inline tension dynamometer to report sex-stratified normalized torque values (Nm/kg) and adduction:abduction ratios. Maximal isometric hip abduction and adduction peak forces were measured during a single session and normalized to body mass and moment arm length. Participants included 35 male runners (18.9±0.90 y) and 26 female runners (19.0±1.1 y). Normalized hip torque (Nm/kg) (right/left) for men was: abduction (1.82±0.38/1.84±0.36), adduction (1.98±0.43/1.70±0.41), and adduction:abduction ratio (1.10±0.18/0.92±0.12). Normalized hip torque (Nm/kg) (right/left) for women was: abduction (1.45±0.25/1.43±0.27), adduction (1.40±0.22/1.34±0.28), and adduction:abduction ratio (0.98±0.14/0.95±0.15). By accounting for joint moment arm length and body mass while minimizing measurement variability through rigid external fixation, this study establishes sex-stratified normative isometric hip strength reference profiles in healthy collegiate distance runners to support baseline strength profiling and standardized clinical screening.

## Introduction

Running-related injuries (RRIs) are common in collegiate cross-country athletes, with reported seasonal incidence rates ranging from approximately 30% to 70%. 1​ 2​ ​3​ Despite decades of investigation, many identified risk factors for RRIs are either nonmodifiable (e.g., sex and prior injury history) or difficult to operationalize reliably within routine preseason screening environments. 1​ 2​ ​3​ As a result, there remains continued interest in identifying modifiable physical characteristics that can be feasibly assessed and monitored within applied sport and clinical settings.

Hip strength testing is frequently incorporated into preseason assessments and rehabilitation programs for runners, based on the proposed role of the hip musculature in controlling the pelvic position and frontal-plane lower-extremity mechanics during stance. The hip abductors contribute to pelvic stabilization during single-limb support, while the hip adductors assist in mediolateral load transfer and force distribution across repeated gait cycles. 4​ 5​ ​6​ Several studies have examined associations between hip strength and RRI, with mixed findings. Systematic reviews indicate that meaningful associations are most consistently observed for iliotibial band syndrome, where reduced hip abductor strength appears more prevalent, while relationships with patellofemoral pain, medial tibial stress syndrome, tibial bone stress injury, and Achilles tendinopathy have not been clearly established. 7​

A persistent challenge in the hip strength literature is the lack of standardized, clinically scalable measurement techniques. Isokinetic dynamometry has been widely used in research settings and provides torque-based outcomes under controlled conditions; however, its designation as a criterion or reference standard should be interpreted cautiously when used as a comparator for portable strength assessments. Systematic reviews examining the criterion validity of portable dynamometry relative to isokinetic systems report highly variable correlations ( *r* ≈0.40–0.93), reflecting substantial heterogeneity across study designs. 8​ Importantly, these discrepancies are likely driven less by fundamental disagreement between measurement constructs and more by variability in methodological execution within portable testing environments. Differences in joint positioning, stabilization strategies, contraction mode, force vector alignment, and normalization procedures can meaningfully influence recorded outputs and limit direct comparability across devices. Consequently, inconsistencies between isokinetic and field-based strength measures can be understood as methodological execution issues rather than evidence that portable dynamometry is inherently unsuitable for estimating joint-level strength.

Hand-held dynamometry (HHD) improves feasibility relative to isokinetic testing but remains highly dependent on the testing configuration. Evidence syntheses report mostly inconsistent criterion validity of HHD when compared with isokinetic dynamometry, supported by low- to moderate-quality evidence. 8​ Examiner strength limitations and inadequate stabilization further contribute to variability, particularly when testing trained or physically developed athletes. 9​ These limitations underscore the importance of testing methodology rather than device selection alone. This is particularly relevant in collegiate athletes, where the muscle force often exceeds the manual stabilization capacity of the examiner, leading to underestimated strength values.

Externally fixated dynamometry addresses several of these methodological concerns by minimizing examiner-related confounding and improving mechanical consistency across trials. Prior work has demonstrated that rigid fixation and belt-stabilized testing configurations improve consistency of force application during hip strength assessment. 10​ ​11​ Inline tension dynamometers, when combined with external fixation, allow for standardized joint positioning, precise alignment of the applied force vector, and consistent loading across participants. Accordingly, testing approaches that emphasize rigid external fixation, standardized positioning, and torque-based outcomes may improve within-study consistency and cross-study interpretability when portable dynamometry is used in applied settings.

Many studies report hip strength using force (N) or body mass normalized force (N/kg), which does not account for individual differences in segment length and joint lever arm. Because joint torque reflects the product of force and moment arm, torque-based metrics provide a more biomechanically representative estimate of joint level strength capacity. Normalizing torque to body mass (Nm/kg) further enhances comparability across athletes with differing anthropometrics and may be particularly relevant in distance runners, where absolute force outputs are modest but relative strength capacity varies. While normalization to body mass improves interpretability relative to force-only metrics, optimal normalization approaches (including allometric scaling) remain debated and are beyond the scope of the present study. 12​

Runner-specific hip strength reference values remain limited and are influenced by testing modality, contraction type, fixation strategy, and population characteristics. Eccentric hip abduction (ABD) strength values have been reported in novice runners, with sex-specific differences observed. 13​ In collegiate cross-country populations, normative isometric hip strength values have been described using HHD, with relationships to prior injury history explored. 14​ More recently, investigations in female collegiate cross-country runners have examined relationships among hip strength, running biomechanics, and injury occurrence, highlighting continued interest in runner-specific profiling alongside substantial methodological variability across studies. 15​ Collectively, differences in testing procedures and reporting units limit direct comparisons and underscore the need for additional population-specific data sets derived from methodologically consistent protocols.

The hip adduction-to-abduction (ADD:ABD) ratio has been proposed as a clinically appealing summary metric of frontal-plane strength balance; however, its relationship with injury risk or performance in distance runners remains understudied. Additionally, recent evidence suggests that there is no relationship between this ratio and the development of groin pain in multidirectional sports. 16​ Accordingly, ratio-based outcomes should be interpreted descriptively rather than diagnostically until further evidence establishes their relevance in this population. 16​

Therefore, the purpose of this study was to describe preseason hip ABD and adduction (ADD) strength in Division III cross-country athletes using an externally fixated inline tension dynamometer and to report sex-stratified normalized torque values (Nm/kg) and ADD:ABD ratios. By emphasizing standardized positioning, rigid external fixation, and torque-based normalization, this study aims to extend the runner-specific evidence base and provide clinically interpretable reference values using a biomechanically grounded and scalable testing methodology.

## Methods

### Participants

This study was approved by the Institutional Review Board at George Fox University (Approval No. 2243025) and all participants provided written informed consent prior to data collection. Participants were 61 healthy Division III collegiate cross-country runners who competed for George Fox University during the 2025 season. Data collection occurred during the pre-season before any competitions had taken place. Informed consent, demographics, and injury history were obtained using an original survey via REDCap (Research Electronic Data Capture; Nashville, Tennessee, USA). REDCap is a secure, web-based software platform designed to support data capture for research studies. Height and weight were obtained using a stadiometer and standard scale, respectively.

### Strength assessment

All participants had a one-time hip strength assessment for ABD and ADD within a battery of preseason assessments. Other assessments included a biomechanical running analysis on a treadmill, vertical and standing long jump analysis using force plates, and an isometric hip extensor strength assessment using a Biodex isokinetic dynamometer. Hip ABD and ADD strength was assessed by a single licensed physical therapist who was blinded to results from the REDCap survey and other assessments performed on the same day.

**Fig. 1:**
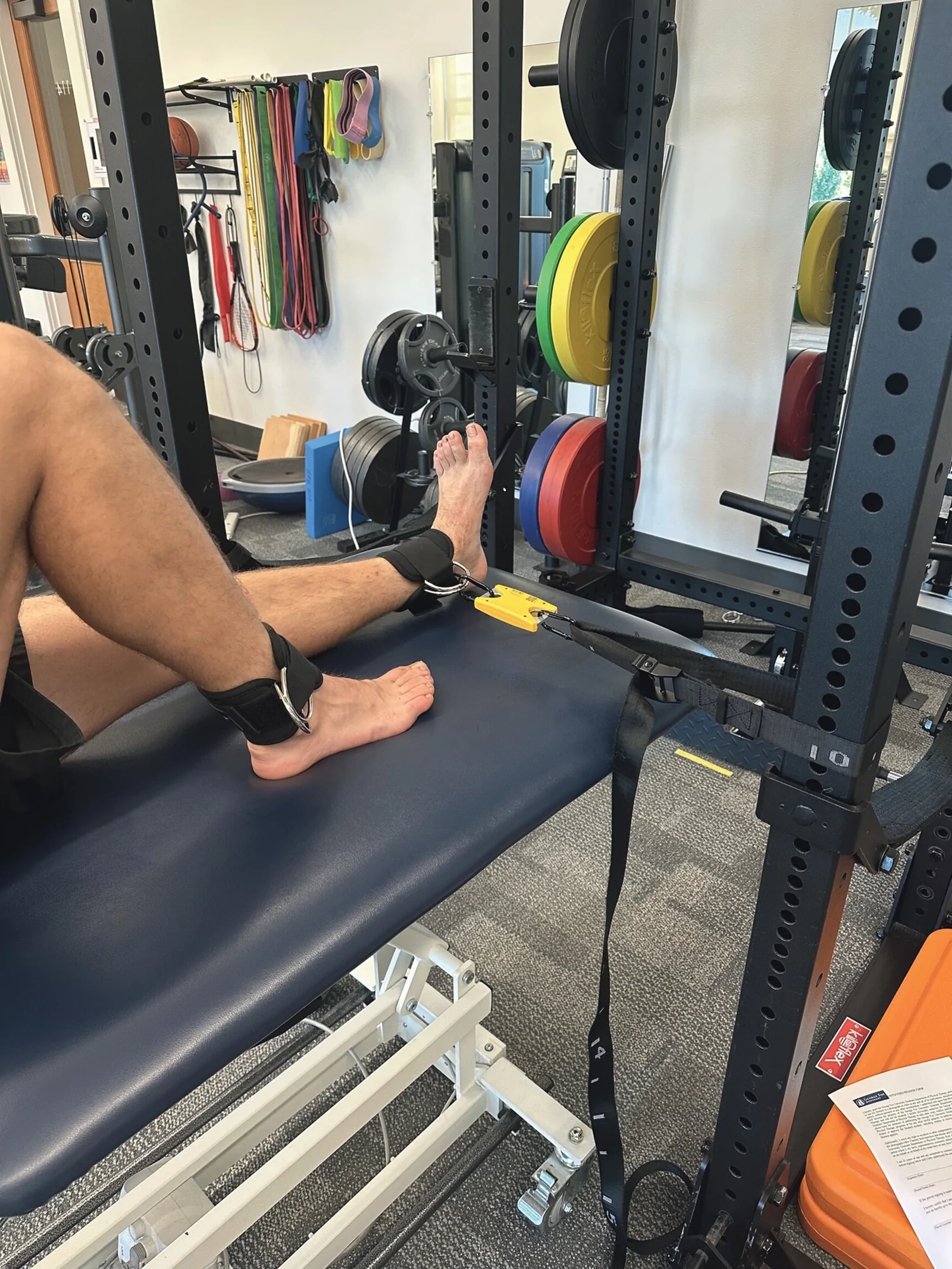
Hip abduction strength assessment using a dynamometer with a fixed strap: male.

**Fig. 2:**
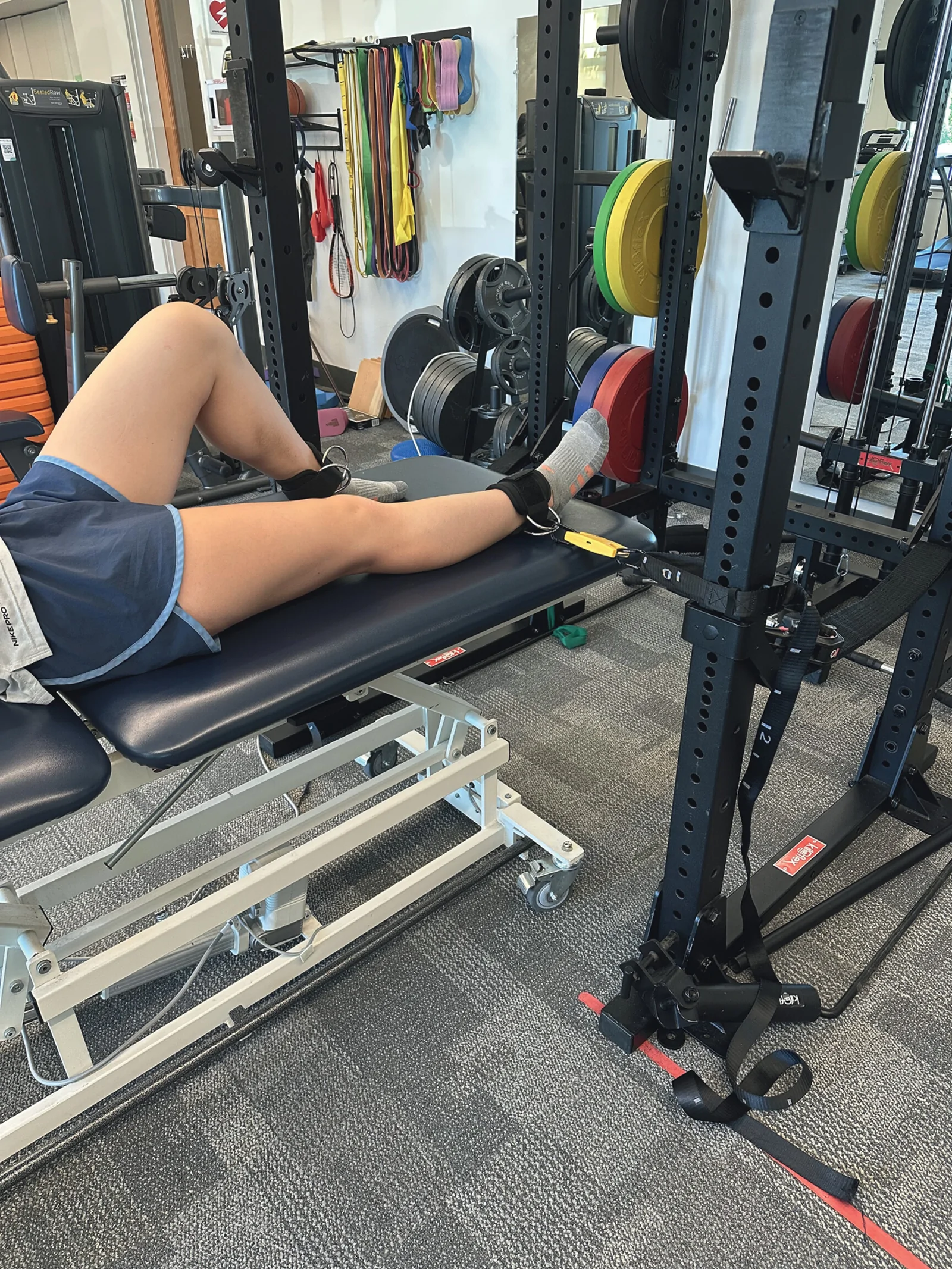
Hip adduction strength assessment using a dynamometer with a fixed strap: Female.

The hip strength assessment was performed in the supine position for both hip ABD and ADD near the optimal force–length angles for the hip in the frontal plane. A gymnastic strap with an ankle cuff was placed perpendicular to the point 5 cm superior to the inferior aspect of the medial malleolus ( Fig. 1 ). For ABD, participants were placed in slight ADD, and once the slack was removed, they were at 0° (neutral hip position) with the contralateral limb in knee flexion ( Fig. 1 ). For hip ADD, the participant started in slight ABD before the slack was removed and began testing in a neutral hip position with the contralateral limb in knee flexion ( Fig. 2 ). For both hip ABD and ADD, the hip and the knee were in the neutral position in the sagittal plane. The moment arm for ABD and ADD was measured from the anterior superior iliac spine to 5 cm superior to the inferior portion of the medial malleolus, which was at the midpoint of the ankle cuff ( Fig. 1 ).

Data were collected using a Tindeq Progressor 500 dynamometer (Trondheim, Norway) utilizing a fixed strap. Samples were collected at a frequency of 80 Hz and the design load was 5000 N (500 kg). Three trials were performed resulting in the maximum value for both ABD and ADD with a 10-second rest between each trial on each side before moving to the opposite side. The order of testing for ABD and ADD was counterbalanced for each participant.

Participants were cued at the beginning of the assessment to “slowly build up to maximal force within 5 seconds” and were given a single practice trial to familiarize them with the protocol. They were not given the ability to look at the iPad screen for force values during assessment. Participants completed three maximal voluntary contractions, and the highest peak force was used to calculate torque. Body mass was measured in kilograms and the moment arm was measured in centimeters and then converted to meters.

### Data analysis

All incomplete data were removed before being imported into the Statistical Package for Social Sciences (SPSS) version 30 (Chicago, Illinois, USA). Survey data were downloaded from REDCap to a CSV file with labels. Peak torque (Nm) was calculated using the absolute peak force recorded across all three trials for both hip ABD and ADD, multiplied by the moment arm. Peak torque was then normalized to body mass in kilograms (Nm/kg), and the peak ADD:ABD ratio was calculated for both right and left limbs across all participants. While standard strength-testing protocols dictate using only the single maximum trial to derive primary torque outcomes, all three trials were utilized to assess repeatability. Specifically, the coefficient of variation (CV) was calculated across all three trials for each participant and testing condition. These individual CV values were then averaged across the sample by sex to determine group-level reliability ( Table 2 ). Reliability was categorized as high (CV<10%), moderate (CV=10–20%), and low (CV>20%). 17​

## Results

A total of 61 healthy Division III cross-country runners (35 men and 26 women) completed the REDCap survey and hip strength assessment. Male runners had a mean age of 18.9±0.90 (±standard deviation) years, body mass of 68.7±7.9 kg, and body mass index (BMI) of 21.9±1.9 kg/m ^2^ . Female runners had a mean age of 19.0±1.1 years, body mass of 59.1±5.6 kg, and BMI of 21.8±1.9 kg/m ^2^ . The male runners were identified as 74% Caucasian, 14.3% multiracial, 5.7% Asian/Pacific Islander and 5.7% Hispanic. The female runners were identified as 88.5% Caucasian, 7.7% multiracial and 3.8% Hispanic ( Table 1 ).

**Table:** **Table 1** Demographic data

	Male, *n* =35	Female, *n* =26
Age (y)	18.9±0.90	19.0±1.1
Body mass (kg)	68.7±7.9	59.1±5.6
BMI (kg/m ^2^ )	21.9±1.9	21.8±1.9
Race/ethnicity		
White/Caucasian	26/35=74.3%	23/26=88.5%
Multiracial	5/35=14.3%	2/26=7.7%
Asian/Pacific Islander	2/35=5.7%	0/26=0%
Hispanic	2/35=5.7%	1/26=3.8%

Abbreviation: BMI, body mass index.

Note: Values are presented as mean±standard deviation.

**Fig. 3:**
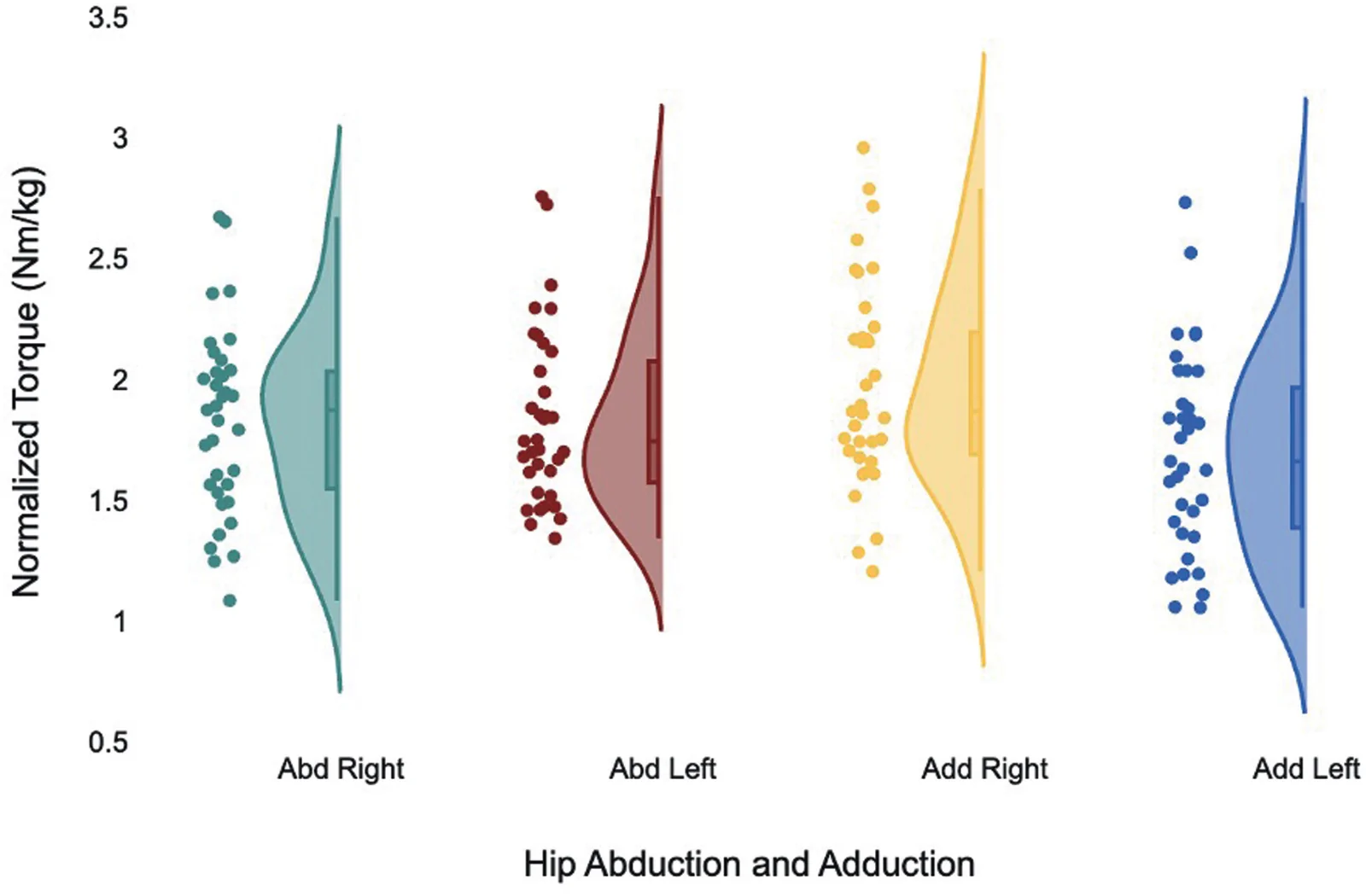
Normalized torque for male hip abduction and adduction.

**Fig. 4:**
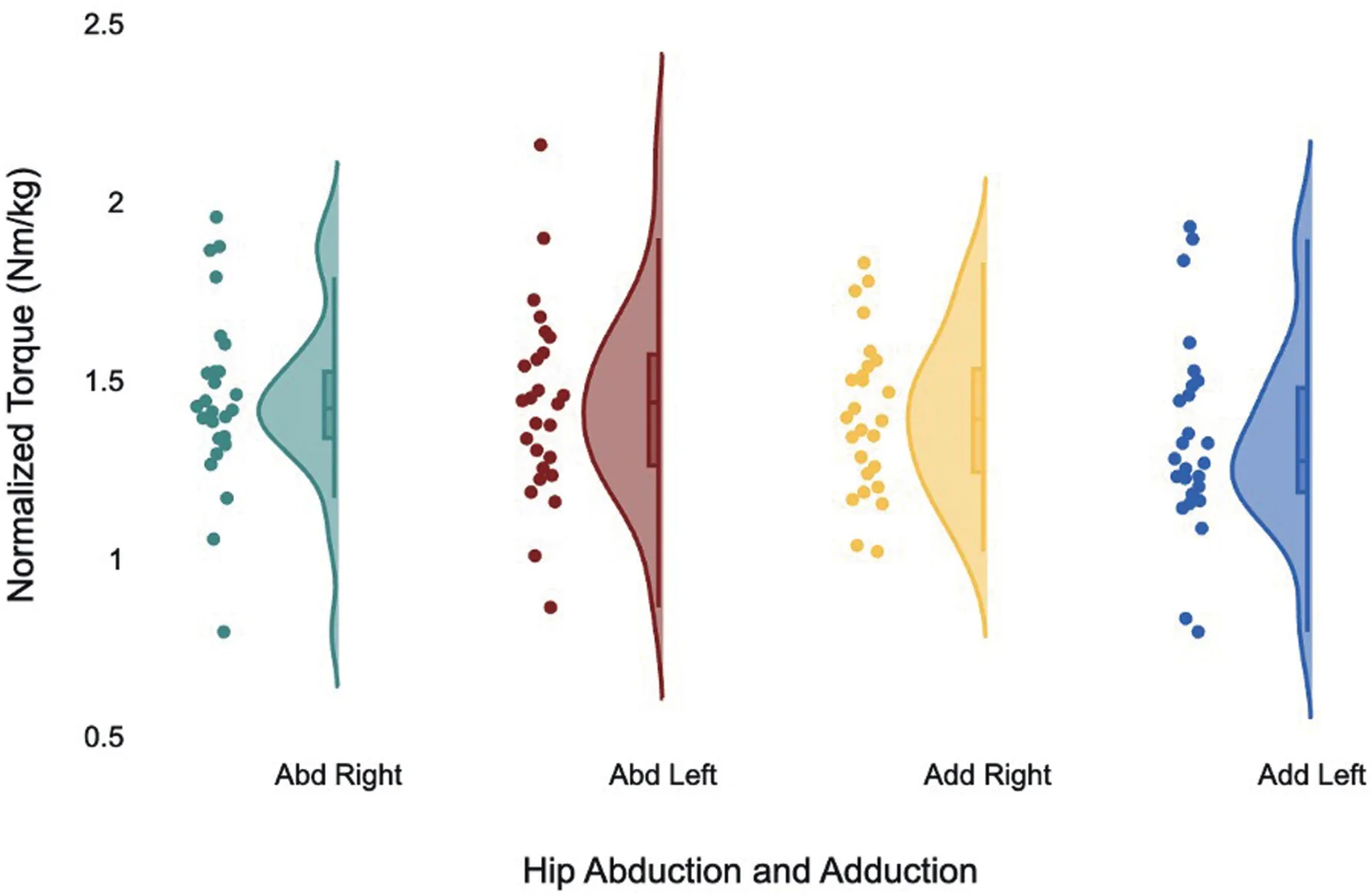
Normalized torque for female hip abduction and adduction.

Descriptive statistics of normalized peak hip torque for ADD, ABD and ADD:ABD ratio are described in Table 2 for women and men. The CV for the male runners was between 16.76% and 21.59% and that of female runners was 15.54% to 20.64%. Normalized torque for ABD and ADD was visualized using a raincloud plot for both men ( Fig. 3 ) and women ( Fig. 4 ).

**Table:** **Table 2** Descriptive statistics (mean, SD, minimum and maximum and CV) of the maximal hip isometric strength test for abduction and adduction. Normalized Torque (Nm/kg) for preseason female and male cross-country runners. All normalized torque values are expressed in Nm/kg

Measure/movement	Sex	Mean±SD	Min	Max	CV (%)
Hip abduction (right; Nm/kg)	Female	1.45±0.25	0.80	1.96	17.31
Hip abduction (right; Nm/kg)	Male	1.82±0.38	1.09	2.67	20.99
Hip abduction (left; Nm/kg)	Female	1.43±0.27	0.86	2.16	18.87
Hip abduction (left; Nm/kg)	Male	1.84±0.36	1.34	2.76	19.67
Hip adduction (right; Nm/kg)	Female	1.40±0.22	1.02	1.83	15.54
Hip adduction (right; Nm/kg)	Male	1.98±0.43	1.21	2.96	21.59
Hip adduction (left; Nm/kg)	Female	1.34±0.28	0.80	1.93	20.64
Hip adduction (left; Nm/kg)	Male	1.70±0.41	1.06	2.73	16.76
Hip adduction:abduction ratio (right)	Female	0.98±0.14	0.73	1.47	–
Hip adduction:abduction ratio (right)	Male	1.10±0.18	0.78	1.73	–
Hip adduction:abduction ratio (left)	Female	0.95±0.15	0.72	1.31	–
Hip adduction:abduction ratio (left)	Male	0.92±0.12	0.64	1.26	–

Abbreviations: CV, coefficient of variation; SD, standard deviation.

## Discussion

Our findings establish normative hip torque values in a Division III collegiate population using a novel, externally fixated methodology. Notably, our reported ABD torque values were higher than those previously reported using non-fixated HHD. This likely reflects the elimination of tester-strength bias and the inclusion of moment-arm normalization, which provides a more accurate representation of true joint-level capacity than force-only metrics.

The hip ABD normalized torque values observed in our sample were comparable or higher than those reported across all available running-based populations, although the pattern of differences is not entirely consistent with what would be expected based on the testing method alone. The mean hip ABD torque in the present sample ranged from 1.43 to 1.45 Nm/kg in women and 1.82 to 1.84 Nm/kg in men. Baggaley and colleagues found a mean hip ABD torque of 0.86 Nm/kg in female recreational runners, far below our current findings. 18​ In line with our findings, Radzak et al reported an isometric hip ABD torque of 1.78 Nm/kg in men. 19​ Isokinetic testing, which is generally expected to yield lower torque values than isometric testing due to the velocity-dependent nature of force production, was used by Venable et al (90°/s), Ford et al. (120°/s), and Sever et al. (60°/s). 15​ 20​​ ​21​ Venable and colleagues reported a mean hip ABD torque of 1.57 Nm/kg in women, although the population included Division I and Division II collegiate runners. 15​ Ford and colleagues reported much lower hip ABD torque values of 1.0 Nm/kg in women and 1.30 Nm/kg in men, consistent with what is expected at faster isokinetic speeds. 20​ The mean hip ABD torque values in the current study were notably higher than those of Sever et al., who reported 1.27 Nm/kg in elite male runners. 21​ Ramskov and colleagues employed a hand-held dynamometer using eccentric break testing without external fixation in novice runners, reporting a mean of 1.41 Nm/kg in women and 1.62 Nm/kg in men. 13​ Eccentric contractions are generally capable of producing greater force than isometric contractions, and on that basis one would expect the Ramskov values to exceed ours. The fact that our findings are comparable or higher than their eccentric values likely reflects meaningful methodological limitations of the hand-held dynamometer approach, particularly the absence of external fixation. This has been documented as a concern in hip abductor strength testing and may account for the lower values observed despite the eccentric contraction mode. Differences in athletic training status (i.e. novice runners versus trained collegiate athletes) may also contribute to this disparity.

The mean hip ADD torque in the present sample ranged from 1.34 to 1.40 Nm/kg in women and 1.70 to 1.98 Nm/kg in men. To our knowledge, normalized isometric adductor torque has not been reported in cross-country running populations. Running-based field sport populations have demonstrated significantly higher adductor strength, but based on sport-demands it is inappropriate to make these comparisons. 22​ ​23​

The ADD:ABD in the current study ranged from 0.95 to 0.98 in women and 0.92 to 1.10 in men. The larger discrepancy in men was the result of a lower group average in left adductor torque. While these values are comparable to those published in the previous literature on multi-directional sports, caution should be taken when comparing the populations. 16​ The physical demands of long-distance running are notably different than those of the sports with available data for comparison, which underscores the novelty of this investigation. Additionally, with this recent data not supporting the idea that there is an optimal ratio to reduce the risk of injury, we advocate for assessing normalized torque to better contextualize the athlete’s capacity.

Collectively, these comparisons underscore the challenge of benchmarking hip strength across studies that differ in contraction type, collection method, angular velocity, and fixation method. Despite these limitations, the values reported here may serve as a useful isometric reference for Division III collegiate male and female cross-country runners. Standardization of hip strength testing protocols across future studies is needed to improve comparability and establish robust normative data for competitive running populations.

The testing setup used in this study, an inline tension dynamometer paired with a fixed ankle cuff and strap, was both clinically accessible and time-efficient. Notably, all assessments were conducted by a single physical therapist, eliminating interrater variability and ensuring consistency throughout each trial. The inline orientation of the dynamometer allowed for alignment with the direction of an applied force, further improving accuracy and reproducibility. The CV of peak torque values ranged from 15.54 to 21.59%, with three of eight conditions exceeding the 20% threshold. The CV values could be due to biological diversity reflecting varying levels of preseason fitness, rather than the measurement error, given the rigid fixation method.

Unlike isokinetic testing, which is frequently cost-prohibitive, technically complex, and poorly suited to field or outpatient environments, our approach offers a scalable alternative without compromising data quality. Given the central role of ABD and ADD in frontal plane control, pelvic stabilization, and lower extremity injury prevention, a rapid, reliable, and reproducible strength assessment is essential for clinicians operating in sport, orthopedic, or rehabilitation contexts. 6​ 24​​ ​25​ This study presents a viable framework for incorporating externally fixated torque-based testing into real-world clinical settings.

This study addresses a gap in normative strength data by examining ABD and ADD torque in Division III collegiate male and female runners using a standardized, externally fixated protocol. Much of the existing literature focuses on different running populations and uses heterogeneous testing methodologies, which can obscure meaningful sex-based differences in strength and biomechanics. By isolating this population and minimizing test variability, our data provide much-needed reference values to inform return-to-play guidelines, strength benchmarks, and injury risk profiles for male and female collegiate runners. Future investigations should seek to build upon these findings through larger sample sizes and broader stratification by sport, age, race/ethnicity, gender identity, and injury history. Such efforts will be essential to advancing equity and clinical utility in musculoskeletal assessment.

## Conclusions

Normalized hip strength values for ABD and ADD, accounting for joint moment arm and body mass, were established in collegiate cross-country runners using an externally fixated inline tension dynamometer. These values provide reference profiles for clinical assessment, screening, and return-to-sport decision-making, and demonstrate a clinically practical methodology for comprehensive hip strength testing.

## Practical implications

The use of rigid external fixation and torque-based normalization (Nm/kg) provides a more accurate and scalable assessment of hip strength than raw force measurements.
These values serve as a preseason benchmark for clinicians to identify deficits and guide return-to-sport testing.
This study establishes sex-stratified normative hip torque values for collegiate cross-country runners using an externally fixated inline dynamometer.
Normalized ABD torque values were higher than values typically reported via non-fixated HHD.
